# The U-shaped association of fasting plasma glucose to HbA1c ratio with mortality in diabetic and prediabetic populations: the mediating role of systemic immune-inflammation index

**DOI:** 10.3389/fendo.2025.1465242

**Published:** 2025-01-27

**Authors:** Ming Yang, Qing Shangguan, Guobo Xie, Guotai Sheng, Jingqi Yang

**Affiliations:** Department of Cardiovascular Medicine, Jiangxi Provincial People’s Hospital, The First Affiliated Hospital of Nanchang Medical College, Nanchang, Jiangxi, China

**Keywords:** fasting plasma glucose to glycated hemoglobin ratio, mortality, immunity, inflammation, diabetes

## Abstract

**Backgrounds:**

This study aimed to assess the association between fasting plasma glucose to glycated hemoglobin (FPG/HbA1c) ratio and mortality and to explore the mediating role of immunity and inflammation in diabetic and prediabetic populations.

**Methods:**

Our analysis included 10,267 participants with prediabetes or diabetes from the NHANES (1999-2018). The association between the FPG/HbA1c ratio and all-cause and cardiovascular(CVD) mortality was assessed using multivariate Cox proportional hazards models, restricted cubic splines(RCS), two-piecewise Cox proportional hazards models and sensitivity analysis. Mediation analysis was conducted to evaluate the systemic immune-inflammation index (SII) as a potential mediator.

**Results:**

Over a median follow-up of 103 months, there were 535 CVD deaths and 1918 all-cause deaths. After multivariate adjustment, a U-shaped relationship was observed between the FPG/HbA1c ratio and both CVD and all-cause mortality, with threshold points at 1.080 and 1.013, respectively. Below the thresholds, the FPG/HbA1c ratio was negatively associated with CVD mortality (HR:0.200, 95% CI: 0.072, 0.559) and all-cause mortality(HR: 0.242, 95% CI: 0.118, 0.494). Above the thresholds, the ratio was positively associated with CVD mortality (HR=3.691, 95% CI: 2.011, 6.772) and all-cause mortality (HR=3.025, 95% CI: 2.279, 4.016). Mediation analysis revealed that SII mediated 19.02% of the association with CVD mortality and 8.86% with all-cause mortality (P < 0.05).

**Conclusions:**

In the prospective cohort, the FPG/HbA1c ratio demonstrated a U-shaped association with mortality in diabetic and prediabetic adults, with SII playing a significant mediating role. These findings suggest that interventions targeting immunity and inflammation may improve clinical outcomes in these populations.

## Introduction

Diabetes mellitus is a metabolic disorder characterized by prolonged high blood sugar levels (1). It is a major public health concern worldwide, with an estimated 9.3% of the adult population affected as of 2019, according to the International Diabetes Federation (2). Diabetes not only affects individuals' quality of life but also significantly contributes to the burden of cardiovascular diseases (CVD) which is the leading cause of death in this population (3).

Identifying predictive factors for mortality in diabetes mellitus (DM) is crucial due to the complex nature of the disease. The interplay between stress hyperglycaemia and adverse outcomes in diabetic and pre-diabetic populations has become an area of increasing interest in cardiovascular and endocrine research (4, 5). Stress hyperglycaemia, characterised by a transient increase in blood glucose levels in response to acute stress. This phenomenon is particularly relevant in the context of diabetes or pre-diabetes, where the balance between glucose homeostasis and stress responses is often disrupted, leading to increased susceptibility to complications (6, 7). It is postulated that the increase in glucose levels during stress not only reflects the body's immediate metabolic response, but may also contribute to the long-term cardiovascular risks faced by these patients (8).

Recent research has emphasised the importance of the FBG/HbA1c ratio as a marker of stress hyperglycemia (9). This ratio reflects the body's glucose response to acute stressors and is indicative of the stress-induced hyperglycemic state. The ratio of FBG to HbA1c captures the relationship between an immediate glycemic measure and a long-term glycemic indicator. It may provide a new way to assess the risk of adverse outcomes in diabetes management. In addition, the stress response activates multiple immune and inflammatory pathways, leading to an increased release of inflammatory factors such as cytokines and chemokines (10, 11). These factors not only directly damage the cardiovascular system, but may also exacerbate the adverse effects of hyperglycaemia by affecting insulin signalling and glucose metabolism (12). Therefore, in diabetic population, we speculated that stress hyperglycaemia may affect their prognosis through immune and inflammatory pathways.

The present study aims to investigate the association between the FBG/HbA1c ratio and mortality outcomes in individuals with diabetes or pre-diabetes, with a particular focus on the potential mediating role of systemic immune and inflammatory. By examining these relationships, we aim to improve our understanding of the mechanisms underlying the association between stress hyperglycaemia and mortality and to identify potential therapeutic targets for intervention.

## Methods

### Study population

The study included participants from the National Health and Nutrition Examination Survey (NHANES, https://wwwn.cdc.gov/nchs/nhanes/Default.aspx), a cross-sectional survey designed to provide a nationally representative sample of the US population. The NHANES is a program conducted by the Centers for Disease Control and Prevention (CDC) designed to assess the health and nutritional status of adults and children and is based on a complex, multistage, probability sampling design that allows it to represent the US population. We analyzed the data from the last 10 cycles (1999–2018) of the diabetes and pre-diabetes population.

**Figure 1:**
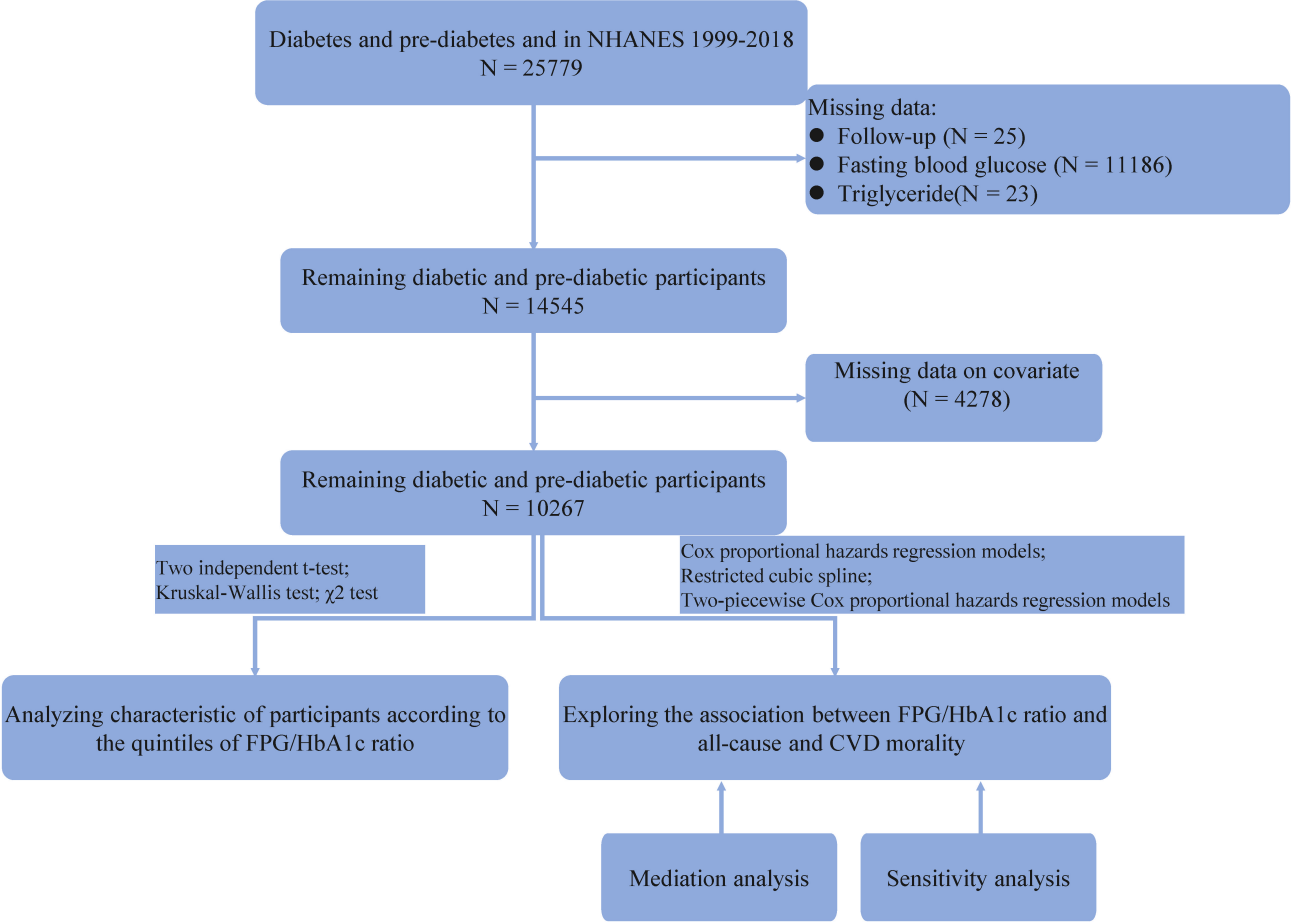
Study flowchart.

Diabetes was defined by self-reported diagnosis by a physician or health care professional, or fasting blood glucose (FBG) ≥ 7mmol/L, or HbA1c level ≥ 6.5%. Pre-diabetes was defined as self-reported pre-diabetes status by a physician or health care professional, or FBG between 5.6mmol/L and 6.9mmol/L or HbA1c between 5.7% and 6.4% (13). After screening, 10267 people with diabetes or pre-diabetes were finally included (**Figure 1**).

### Study variables

The fasting plasma glucose (FPG) /HbA1c ratio was calculated using the formula (14): FPG (mmol/L)/ HbA1c (%). Fasting plasma samples (at least 8 hours or more but less than 24 hours) were collected and analyzed using standard laboratory techniques. FPG levels are determined using enzymatic assays on automated chemistry analysers. HbA1c is determined by high-performance liquid chromatography (HPLC) or ion-exchange chromatography, which separates different forms of haemoglobin based on differences in electrical charge. All HbA1c measurements in our study were performed in laboratories certified by the National Glycohemoglobin Standardization Program (NGSP) and standardized to the Diabetes Control and Complications Trial (DCCT) assay, ensuring the accuracy and reliability of our results. All patients were divided into five groups (Q1, Q2, Q3, Q4 and Q5) according to the quintiles of the FPG/HbA1c ratio, where Q1 represents the lowest levels as well as the reference group.

Immunity and Inflammation were used as a mediating variable, as reflected by the Systemic Immune Inflammation (SII) index. It is a new comprehensive inflammatory biomarker based on neutrophil, lymphocyte, and platelet counts, which accurately reflects the immunity and inflammation in a wide range of conditions. In this study, we calculated the SII index for each participant as follows (15): SII index (×10^9^/L) = neutrophil count (×10^9^/L)/lymphocyte count (×10^9^/L) × platelet count (×10^9^/L). Blood cell counts were measured using an automated haematology analyser.

Mortality data were obtained by linking NHANES participants to the National Death Index (NDI), which provides information on date and cause of death from the National Centre for Health Statistics through 31 December 2019. We defined all-cause mortality as any death recorded in the NDI, while cardiovascular mortality was identified using the International Classification of Diseases, 10th Revision (ICD-10) codes for heart diseases (I00-I09, I11, I13, I20-I51).

### Covariates

The information on age, gender, race, education, family poverty income ratio, smoking status, and alcohol intake frequency were collected using standardized interview questionnaires. Race were classified as non-Hispanic White, non-Hispanic Black, Mexican American or Hispanic, or other. educational attainment categorized as less than high school, high school diploma or equivalent, and college education or higher, as well as the family poverty income ratio, which was stratified into ≤1, >1 to ≤3, and >3. Participants were classified as never smoked, current smoker, and former smoker according to their responses about smoking at least 100 cigarettes during their lifetime and whether they were currently smoking. The frequency of alcohol consumption was determined on the basis of the 24-h dietary recall of participants and they were categorised as never, daily or nearly daily, 3 to 4 times per week, 1 to 2 times per week, and less than once per week. The measurement of systolic blood pressure (SBP) and diastolic blood pressure (DBP) was conducted using a sphygmomanometer, typically under resting conditions, with the average of multiple measurements taken to determine the SBP and DBP values.

In addition, rigorous laboratory analyses were performed, including assessment of aspartate aminotransferase (AST, U/L), alanine aminotransferase (ALT, U/L), Serum creatinine(Scr, umol/L), blood urea nitrogen(BUN, mmol/L), total cholesterol (TC, mmol/L), triglyceride (TG, mmol/L), high density lipoprotein (HDL, mmol/L), Low-density lipoprotein(LDL, mmol/L). A detailed description of the laboratory methods used can be found in the Laboratory Methods Documentation section of NHANES(https://www.cdc.gov/nchs/nhanes/index.html ).

### Statistical analysis

R software (version 4.3.0) and Empower software (version 4.1) was used to perform the statistical analyses. Participants were classified into five groups according to quintiles (Q1-Q5) of the FPG/HbA1c ratio. Continuous variables were summarized as mean and standard deviation (SD), while categorical variables were presented as frequency and percentage. The comparison of baseline characteristics across FPG/HbA1c quintile groups was assessed using the Kruskal-Wallis H test or one-way ANOVA for continuous variables and pearson chi-square test for categorical variables. The incidence rates of all-cause mortality and CVD mortality for each FPG/HbA1c quintile group were determined over the follow-up period. To evaluate the prognostic significance of the FPG/HbA1c ratio, Cox proportional hazards regression models were constructed and three models were included: Model 1 was unadjusted, Model 2 was adjusted for age, gender, and race, and Model 3 was adjusted for age, race, education, PIR, BMI, drinking, smoking, SBP, DBP, TG, TC, HDL, LDL, ALT, AST, BUN, Scr. In addition, to investigate the relationship between FPG/HbA1c ratio and all-cause and CVD mortality, we modelled Cox proportional hazards regression using 4-knot(0.05, 0.35, 0.65, 0.95) restricted cubic spline(RCS) and smooth curve fitting (penalised spline method). We apply segmented regression that is using a separate line segment to fit each interval. Log-likelihood ratio test comparing one-line (non-segmented) model to segmented regression model was used to determine whether threshold exists. The inflection point that connecting the segments was based on the model gives maximum likelihood, and it was determined using two steps recursive method. In nonlinear relationships, we used segmented Cox regression models on both side of the effect point to investigate the relationship between FPG/HbA1c ratio and the risk of all-cause and CVD mortality. Finally, the mediation model was constructed to estimate the indirect effects of immunity and inflammation on the relationship between FPG/HbA1c ratio and mortality. The R packages "mediation" (16) were respectively used to estimate the mediation effects of SII index. A total of 1000 repeated simulations were conducted to obtain more accurate estimates of the mediation effects and their confidence intervals, with results presented in terms of effect (β), indirect effect, direct effect, total effect, and P-values.

## Results

### Baseline characteristics of participants

A total of 10267 participants with pre-diabetes and diabetes from NHANES 1999-2018 were included in our study, of whom 54.19% were male and 45.81% were female, with the average age of 55.77 ± 16.42 years. During a median follow-up of 103 months, the occurrence of 535 CVD deaths and 1918 all-cause deaths were found. Baseline characteristics based on FPG/HbA1c ratio quintiles were shown in **Supplementary Table 1**. In different groups of FPG/HbA1c ratio quintiles, gender, age, race, BMI, education, PIR, smoking status, drinking frequency, SBP, DBP, BUN, Scr, AST, ALT, TC, LDL, TG, HDL are significantly different (all P < 0.05). It is noteworthy that the FPG values in the Q0 group were significantly lower, with a mean value of 5.39 mmol/L, while the HbA1c levels were not the highest, at 6.18%. Conversely, the highest Q4 group had the highest FPG values, with a mean of 9.15 mmol/L, and the HbA1c levels were also the highest at 6.70%. This indicates that an elevated FPG/HbA1c ratio is associated with hyperglycemia and relatively higher HbA1c levels. These findings provide a more nuanced perspective on glycemic control among the extremes of our study cohort.

### Relationships of FPG/HbA1c ratio with mortality

We have used three multivariate cox regression models to show the relationship between FPG/HbA1c ratio with mortality in **Table 1**. After adjusting for all variables, the FPG/HbA1c ratio was statistically positively associated with all-cause mortality (HR=1.886, 95% CI: 1.459, 2.439), while it was not statistically significant with CVD mortality (HR=1.460, 95% CI: 0.865, 2.465) in the diabetic and pre-diabetic populations. When the FPG/HbA1c ratio was converted from a continuous variable to a categorical variable(quintiles), CVD mortality was significantly lower in the Q2 group (HR=0.723, 95% CI: 0.534, 0.979) than in the Q1 group(P<0.05), although the P for trend was > 0.05. But in the all-cause mortality, FPG/HbA1c ratio was associated with a decrease in Q1(HR= 0.844, 95% CI: 0.720, 0.989) and Q2 group (HR= 0.845, 95% CI: 0.721, 0.991) and with an increase in Q4 group(HR= 1.204, 95% CI: 1.037, 1.4397) compared to Q1 group(P<0.05), and the P for trend was also < 0.05.

**Table 1:** Relationship between FPG/HbA1c ratio and CVD mortality and all-cause mortality.

	Model 1	Model 2	Model 3
CVD mortality			
FPG/HbA1c ratio	1.690 (1.075, 2.657) 0.023	1.632 (1.021, 2.609) 0.041	1.460 (0.865, 2.465) 0.156
Q0 (0.139-0.957)	1.0	1.0	1.0
Q1 (0.957-1.039)	0.854 (0.653, 1.117) 0.250	0.768 (0.586, 1.006) 0.055	0.819 (0.610, 1.099) 0.184
Q2 (1.040-1.100)	0.774 (0.590, 1.015) 0.064	0.738 (0.560, 0.972) 0.031	0.723 (0.534, 0.979) 0.036
Q3 (1.100-1.185)	0.658 (0.496, 0.874) 0.004	0.685 (0.512, 0.917) 0.01086	0.754 (0.550, 1.033) 0.079
Q4 (1.185-2.998)	1.258 (0.983, 1.610) 0.068	1.120 (0.871, 1.440) 0.37753	1.108 (0.838, 1.464) 0.473
P for trend	0.299	0.385	0.478
All-cause mortality			
FPG/HbA1c ratio	2.125 (1.694, 2.666) <0.001	2.073 (1.638, 2.622) <0.001	1.886 (1.459, 2.439) <0.001
Q0 (0.139-0.957)	1.0	1.0	1
Q1 (0.957-1.039)	0.892 (0.770, 1.033) 0.126	0.813 (0.701, 0.942) 0.006	0.844 (0.720, 0.989) 0.036
Q2 (1.040-1.100)	0.857 (0.741, 0.992) 0.038	0.833 (0.718, 0.967) 0.016	0.845 (0.721, 0.991) 0.039
Q3 (1.100-1.185)	0.852 (0.736, 0.987) 0.033	0.899 (0.774, 1.044) 0.163	0.954 (0.812, 1.121) 0.567
Q4(1.185-2.998)	1.377 (1.205, 1.574) <0.001	1.245 (1.086, 1.426) 0.002	1.204 (1.037, 1.397) 0.015
P for trend	<0.001	<0.001	<0.001

Model 1 adjust for: None. Model 2 adjust for: Age, gender. Model 3 adjust for: Age, gender, race, education, poverty impact ratio, drinking, smoking, systolic blood pressure, diastolic blood pressure, alanine aminotransferase, aspartate transaminase, serum creatinine, blood urea nitrogen, total cholesterol, triglyceride, high density lipoprotein, low density lipoprotein.

FPG/HbA1c, Fasting plasma glucose to glycated hemoglobin; HR, Hazard ratio; CI, Confidence interval.

### The detection of nonlinear relationships

**Figure 2:**
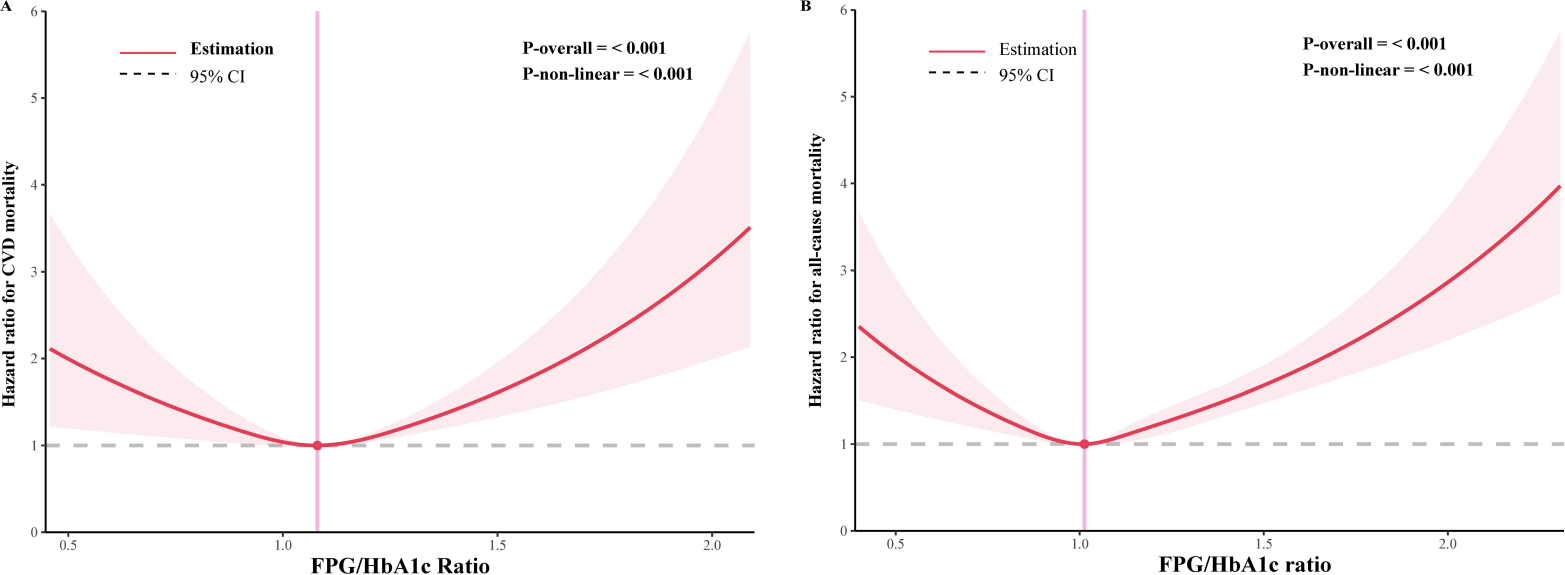
Restricted cubic spline curve for the association of FPG/HbA1c ratio with mortality in diabetic and prediabetic populations. **(A)** Association between FPG/HbA1c ratio and cardiovascular disease mortality. **(B)** Association between FPG/HbA1c ratio and all-cause mortality.

Given that our previous Cox regression analysis suggested a potential non-linear relationship between the FPG/HbA1c ratio and mortality in both pre-diabetes and diabetes, we used RCS and segmented Cox proportional hazards regression models to further investigate the correlation. As shown in **Figure 2**, the RCS analysis of the fully adjusted model revealed that the FPG/HbA1c ratio was associated with both CVD mortality and all-cause mortality (both P < 0.001). Furthermore, the FPG/HbA1c ratio exhibited a U-shaped relationship with CVD mortality as well as all-cause mortality in the diabetic and prediabetic populations. The optimal inflection points were determined to be 1.080 for CVD mortality and 1.013 for all-cause mortality based on the two-piecewise Cox proportional hazards regression models (Both P for non-linear < 0.001). After adjusting for all variables, the risk of CVD and all-cause mortality was reduced by approximately 80.0% (HR=0.200, 95% CI: 0.072, 0.559) and 75.8% (HR=0.242, 95% CI: 0.118, 0.494), respectively, for each unit increase in the FPG/HbA1c ratio before reaching the optimal cut-off point (**Table 2**). Moreover, the risk of CVD and all-cause mortality decreased to the lowest level as the baseline FPG/HbA1c ratio increased to the threshold value. In contrast, when the FPG/HbA1c ratio exceeded the thresholds of 1.080 and 1.013, it was significantly and positively associated with the risk of CVD (HR=3.691, 95% CI: 2.011, 6.772) and all-cause mortality (HR=3.025, 95% CI: 2.279, 4.016, **Table 2**), respectively.

**Table 2:** Threshold effect analysis of FPG/HbA1c ratio on all-cause and CVD mortality.

	Adjusted HR (95% CI)	P-value
CVD mortality		
Inflection point	1.080	
FPG/HbA1c ratio < 1.080	0.200 (0.072, 0.559)	0.002
FPG/HbA1c ratio ≥ 1.080	3.691 (2.011, 6.772)	<0.001
P for Log-likelihood ratio	<0.001	
All-cause mortality		
Inflection point	1.013	
FPG/HbA1c ratio < 1.013	0.242 (0.118, 0.494)	< 0.001
FPG/HbA1c ratio ≥ 1.013	3.025 (2.279, 4.016)	< 0.001
P for Log-likelihood ratio	< 0.001	

Cox proportional hazards models were used to estimate HR and 95% CI. Adjusted for age, gender, race, education, poverty impact ratio, drinking, smoking, systolic blood pressure, diastolic blood pressure, alanine aminotransferase, aspartate transaminase, serum creatinine, blood urea nitrogen, total cholesterol, triglyceride, high density lipoprotein, low density lipoprotein.

### Mediating effect of immunity and inflammation

**Figure 3:**
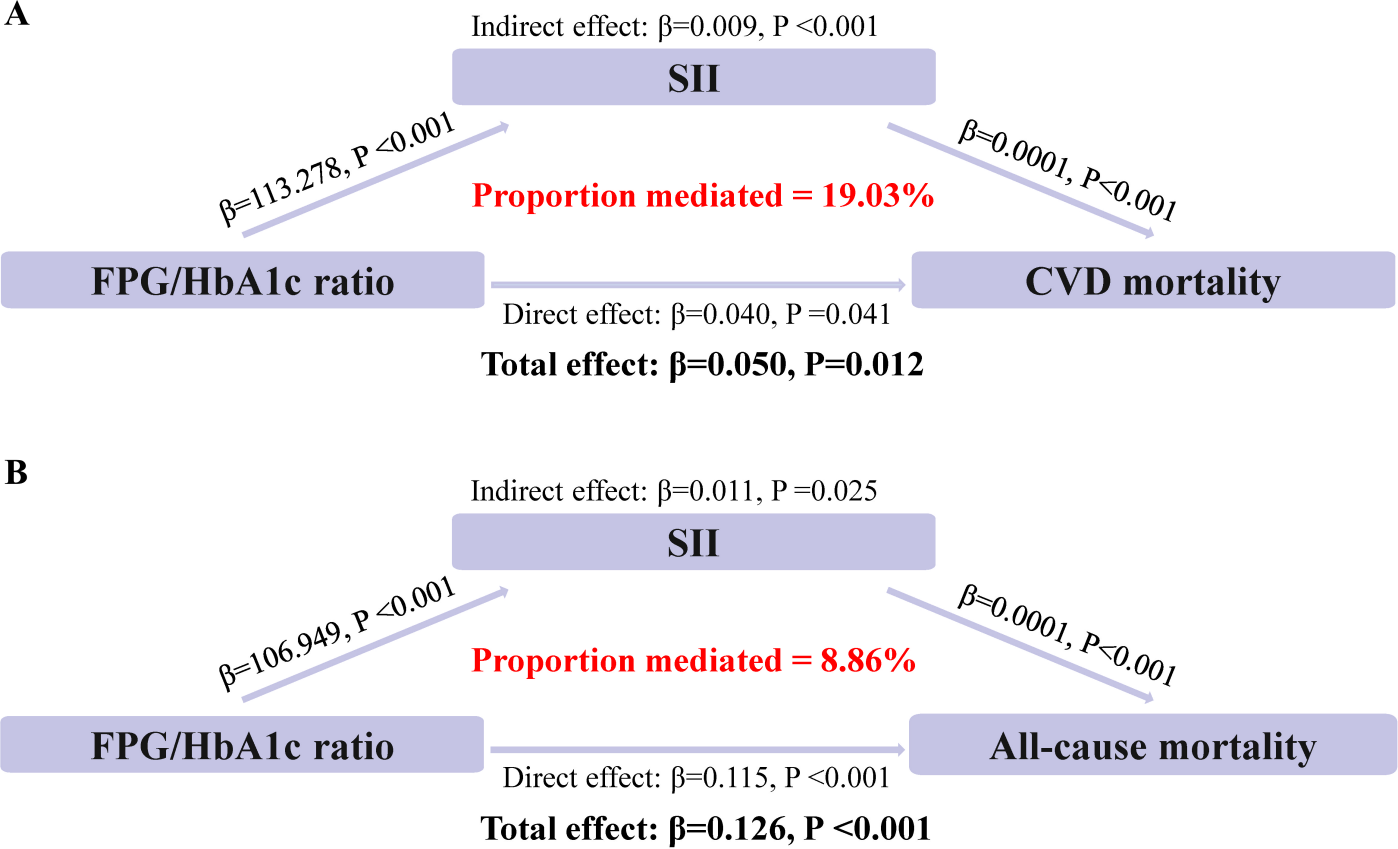
Estimated proportion of the association between FPG/HbA1c ratio and mortality mediated by SII. **(A)** The mediating effect of SII in CVD mortality. **(B)** The mediating effect of SII in all-cause mortality.

Mediation analyses showed that that the SII levels had significant mediating effects on the association between FPG/HbA1c ratio and mortality (**Figure 3**). Notably, SII mediated 19.02% and 8.86% of the association between FPG/HbA1c ratio and CVD mortality (Both P<0.05).

### Sensitivity analysis

To further demonstrate the robustness of our results, we performed the sensitivity analysis in the diabetic and prediabetic populations with prediabetes (sensitivity-1), with diabetes with hypoglycaemic drugs or insulin (sensitivity-2), with CVD (sensitivity-3), with after excluding participants who died within the first 2 years of follow-up (sensitivity-4) and. In the Sensitivity-3, although the P-value for the association between the threshold value of FPG/HbA1c ratio > 1.08 and the CVD mortality was not statistically significant, the trends on either side of the inflection point were consistent with the main results. All other results were broadly consistent with the main analysis, suggesting that the strong robustness of our results (**Supplementary Table 2**).

## Discussion

This study presents novel findings from a comprehensive analysis of NHANES 1999-2018, which revealed that inflammation mediated the association of FPG/HbA1c ratio with CVD and all-cause mortality in the population with diabetes or pre-diabetes for the first time. Our findings suggested a U-shaped association between FPG/HbA1c ratio and mortality, with critical thresholds identified at 1.08 for CVD mortality and 1.013 for all-cause mortality. Furthermore, the immunity and inflammation marker, SII, significantly play a mediating effect in the relationship between FPG/HbA1c ratio and CVD mortality and all-cause mortality.

The standardization of HbA1c measurement has led to its recognition as a reliable diagnostic instrument for assessing long-term blood glucose control (17). It is noteworthy that HbA1c levels are relatively unaffected by transient fluctuations in blood glucose (18). Emerging research indicates that the concept of relative hyperglycaemia, expressed as the ratio of FBG to HbA1c, may offer a more accurate prognostic indicator for critical illnesses than measures of absolute glucose levels (19, 20). A cohort study calculating stress hyperglycemia using the formula: FBG (mmol/L)/[(1.59 × HbA 1c (%)-2.59] (21), to predict all-cause mortality and CVD mortality in prediabetes and diabetes populations, found that this method yielded a U-shaped relationship with all-cause mortality (with an inflection point at 0.87), but an L-shaped relationship with CVD mortality (with an inflection point at 0.93). The inflection points appear to be lower than those calculated in our research, and the U-shaped relationship observed in our study seems to better explain the prognosis of CVD mortality. Within the context of our analysis, stress hyperglycemia is presented as FBG (mmol/L) / HbA1c (%) because it is simpler to calculate and more readily applicable in clinical practice. It has now been demonstrated that the FBG /HbA1c ratio has a significant correlation with acute and critical illness in different populations. Su et al. (22) found that FBG /HbA1c ratio independently predicted 90-day mortality, ICU admission and use of mechanical ventilation during acute illness with extreme hyperglycaemia. In a non-diabetic population, stress hyperglycaemia as measured by the FBG /HbA1c ratio is associated with an increased risk of stroke recurrence and all-cause mortality in acute ischaemic stroke (23). Thus, the FBG /HbA1c ratio can serve as an effective biomarker for stress hyperglycemia.

The association of the FBG /HbA1c ratio with the risk of cardiovascular disease death or all-cause mortality in patients with diabetes and prediabetes remains unclear. A prospective cohort study found that stress hyperglycaemia, as measured by the FBG/HbA1c ratio, was associated with an increased risk of adverse outcomes in the Storke population (9). Gao et al. (24) indicated stress hyperglycaemic ratio is strongly associated with the prognosis of patients with non-obstructive coronary myocardial infarction, especially in diabetic patients. Ding et al. (21) also confirmed that there was a U-shaped association between stress hyperglycaemic ratio and all-cause mortality and an L-shaped association between SHR and cardiovascular mortality. This is similar to our findings. In our study, the U-shaped association between FPG/HbA1c ratio and mortality may reflect the complex interplay between glucose metabolism and health outcomes. On the one hand, in our study, a high FPG/HbA1c ratio is associated with elevated levels of both FPG and HbA1c, with the increase in FPG being greater than that of HbA1c. This suggests poor glycaemic control or an acute stress state, both of which are known risk factors for diabetes-related complications and mortality. Stress hyperglycaemia is a common response to physiological stress when a patient is subjected to acute stress, typically a transient hyperglycaemia during illness (8). Previous studies have demonstrated that stress-induced hyperglycemia can activate the neuroendocrine system, leading to an overproduction of catecholamines and cytokines. This overproduction exacerbates inflammation and oxidative stress, enhances a prothrombotic condition, causes endothelial dysfunction, and damages microcirculatory function, which in turn leads to adverse outcomes (25, 26). On the other hand, a low FPG/HbA1c ratio is associated with lower FPG levels, which may be associated with an increased risk of hypoglycemic events, particularly in patients treated with insulin and hypoglycemic agents. Hypoglycemia can lead to cardiac events and accidents, such as cardiac arrest and syncope, thereby increasing the risk of mortality (27). Additionally, a low FPG/HbA1c ratio may reflect underlying nutritional and metabolic abnormalities, potentially indicating conditions such as malnutrition, frailty or advanced liver disease (28, 29), which are also associated with a poor prognosis.

Immunity and inflammation may be key factors in the FPG/HbA1c ratio and adverse outcomes in diabetic and prediabetic populations. It was all known that immunity and inflammation play vital role in the pathogenesis of both diabetes and cardiovascular disease (30). Furthermore, SII, which has been widely used as a marker of immunity and inflammation, has been demonstrated to predict prognosis in diabetic populations (31). Therefore, SII was used as a mediator in the mediated effect analysis, and it was found that SII accounted for 19.02% and 8.86% of the association between FPG/HbA1c ratio and CVD and all-cause mortality, respectively. SII is a composite variable combining platelet and neutrophil-to-lymphocyte ratios. As a novel inflammatory marker, SII assesses the balance between systemic inflammation and the immune response in an individual, thus providing insights into an individual's inflammatory state and immune system activity.

The hypothalamic-pituitary-adrenal (HPA) axis and the sympathetic nervous system are central to the body's stress response, which can significantly impact glucose metabolism and, by extension, the FPG/HbA1c ratio. When the body is subjected to stress, the HPA axis activates, leading to the release of glucocorticoids, primarily cortisol. Excess cortisol can lead to hyperglycemia by promoting gluconeogenesis in the liver, reducing glucose uptake in adipocytes and skeletal muscles, and inducing insulin resistance (32). Hyperglycemia is significantly associated with adverse prognoses. In addition, the same stress response that dysregulates glucose metabolism also stimulates the release of catecholamines, which can increase immune and inflammatory responses (33). This response is reflected in the SII, which incorporates measures of neutrophils, platelets, and lymphocytes, all of which are influenced by glucocorticoid levels and catecholamine release. An elevated SII, indicative of a heightened inflammatory state, can mediate the relationship between the FPG/HbA1c ratio and mortality. Immunity and inflammation were known to contribute to atherosclerosis, leading to endothelial dysfunction and a prothrombotic state, which are significant risk factors for cardiovascular disease and all-cause mortality (34).

Our study's strengths lie in its large sample size, the use of a well-established cohort with long-term follow-up, and the novel application of mediation analysis to explore the mechanistic pathways linking glucose metabolism, immunity and inflammation, and mortality. These findings contribute to the growing body of evidence that emphasizes the multifactorial nature of diabetes management and the importance of considering immunity and inflammation in addition to glycemic control. While our study provides valuable insights, it also has limitations. Firstly, the FPG/HbA1c ratio was derived from single-timepoint measurements, which may not accurately reflect the fluctuating metabolic states over extended periods. This limitation could influence the prognostic accuracy for diabetic patients. Secondly, as an observational study, it was also subject to potential confounding factors. Although we adjusted for several covariates, residual confounders may still affect the study outcomes. Factors such as dietary patterns, levels of physical activity, and medication adherence, which were not fully accounted for in our analysis, could plausibly act as residual confounders that may influence the study outcomes. Lastly, the generalizability of our findings may be constrained by the fact that our study cohort was predominantly from the U.S. population, which could limit the applicability of our results to other ethnicities or global demographics.

In conclusion, our study provides novel insights into the relationship between the FPG/HbA1c ratio, mortality, and the mediating role of inflammation in a large, nationally representative sample of individuals with diabetes or prediabetes. These findings highlighted the need for a nuanced understanding of glucose metabolism in these populations and suggested potential targets for intervention to improve outcomes. Future research should aim to confirm these findings in other cohorts and to explore the mechanisms underlying the observed associations.

## Data Availability

Publicly available datasets were analyzed in this study. The data of NHANES can be downloaded from the website: https://wwwn.cdc.gov/nchs/nhanes/default.aspx. Further inquiries can be directed to the corresponding author.
