# Supplementary material for: The U-shaped association of fasting plasma glucose to HbA1c ratio with mortality in diabetic and prediabetic populations: the mediating role of systemic immune-inflammation index
Source: Front Endocrinol (Lausanne). 2025 Jan 27;16:1465242. doi: 10.3389/fendo.2025.1465242 (PMC11807827; doi:10.3389/fendo.2025.1465242)
Supplement: Supplementary file 1 [file Table1.docx]

Supplementary Table 1 Baseline characteristics according to FPG/HbA1c ratio quintiles

| FPG/HbA1c ratio quintiles | Q0  (0.139-0.957) | Q1  (0.957-1.039) | Q2  (1.040-1.100) | Q3  (1.100-1.185) | Q4  (1.185-2.998) | P-value |
| --- | --- | --- | --- | --- | --- | --- |
| Gender |  |  |  |  |  | **<0.001** |
| Male | 870 (42.65%) | 1032(50.17%) | 1146(55.79%) | 1246(60.43%) | 1270(61.83%) |  |
| Female | 1170(57.35%) | 1025(49.83%) | 908 (44.21%) | 816 (39.57%) | 784 (38.17%) |  |
| Age (Years) | 56.45±16.12 | 56.95±15.54 | 54.51±16.92 | 53.55±17.15 | 57.39±16.00 | **<0.001** |
| BMI(kg/m^2^) | 30.17±7.33 | 30.65±7.04 | 30.11±6.79 | 30.155±6.87 | 31.110±7.60 | **<0.001** |
| Race |  |  |  |  |  | **<0.001** |
| Mexican American | 288 (14.12%) | 349 (16.97%) | 378 (18.40%) | 411 (19.93%) | 411 (20.01%) |  |
| Other Hispanic | 164 (8.04%) | 178 (8.65%) | 181 (8.81%) | 167 (8.10%) | 172 (8.37%) |  |
| Non-Hispanic White | 673 (32.99%) | 873 (42.44%) | 1005(48.93%) | 1059(51.36%) | 969 (47.18%) |  |
| Non-Hispanic Black | 745 (36.52%) | 446 (21.68%) | 294 (14.31%) | 249 (12.08%) | 361 (17.58%) |  |
| Other Race | 170 (8.33%) | 211 (10.26%) | 196 (9.54%) | 176 (8.54%) | 141 (6.87%) |  |
| Education |  |  |  |  |  | **0.001** |
| Less than high school | 562 (27.55%) | 594 (28.88%) | 530 (25.80%) | 590 (28.61%) | 636(30.96%) |  |
| High or equivalent | 512 (25.10%) | 512 (24.89%) | 544 (26.49%) | 448 (21.73%) | 480(23.37%) |  |
| College or above | 966(47.35%) | 951(46.23%) | 980 (47.71%) | 1024(49.66%) | 938(45.67%) |  |
| PIR |  |  |  |  |  | **0.026** |
| ≤1 | 447 (21.91%) | 394 (19.15%) | 401 (19.52%) | 394 (19.11%) | 435(21.18%) |  |
| >1, ≤3 | 910 (44.61%) | 897 (43.61%) | 908 (44.21%) | 871 (42.24%) | 902 (43.91%) |  |
| >3 | 683 (33.48%) | 766 (37.24%) | 745 (36.27%) | 797 (38.65%) | 717 (34.91%) |  |
| Drinking |  |  |  |  |  | **<0.001** |
| Never | 848 (41.57%) | 741 (36.02%) | 679 (33.06%) | 620 (30.07%) | 754 (36.71%) |  |
| Every day or nearly every day | 339 (16.62%) | 360 (17.50%) | 362 (17.62%) | 369 (17.90%) | 311 (15.14%) |  |
| 3 to 4 times a week | 263 (12.89%) | 263 (12.79%) | 283 (13.78%) | 296 (14.36%) | 271 (13.19%) |  |
| 1 to 2 times a week | 422 (20.69%) | 497 (24.16%) | 532 (25.90%) | 578 (28.03%) | 525 (25.56%) |  |
| Less than once a week | 168 (8.24%) | 196 (9.53%) | 198 (9.64%) | 199 (9.65%) | 193 (9.40%) |  |
| Smoking status |  |  |  |  |  | **<0.001** |
| Never | 1076(52.75%) | 1069(51.97%) | 1052(51.22%) | 1034(50.15%) | 955 (46.50%) |  |
| Now | 439 (21.52%) | 380 (18.47%) | 417 (20.30%) | 398 (19.30%) | 363 (17.67%) |  |
| Former | 525 (25.74%) | 608 (29.56%) | 585 (28.48%) | 630 (30.55%) | 736 (35.83%) |  |
| SBP | 124(113-136) | 124(114-136) | 123(114-136) | 124(115-135) | 127(116-141) | **<0.001** |
| DBP | 70 (62-77) | 71 (63-78) | 71 (63-78) | 71 (64-78) | 71 (63-78) | **0.002** |
| LDL(mmol/L) | 3.08±1.00 | 3.08±0.93 | 3.09±0.94 | 2.98±0.91 | 2.89±0.97 | **<0.001** |
| TC(mmol/L) | 5.12±1.13 | 5.10±1.05 | 5.11±1.06 | 4.99±1.06 | 4.93±1.11 | **<0.001** |
| TG(mmol/L) | 1.35±0.73 | 1.46±0.75 | 1.49±0.75 | 1.51±0.82 | 1.66±0.87 | **<0.001** |
| HDL(mmol/L) | 1.42±0.40 | 1.35±0.38 | 1.33±0.38 | 1.32±0.37 | 1.29±0.42 | **<0.001** |
| ALT(mmol/L) | 24.89±47.22 | 26.70±45.66 | 26.66±19.32 | 27.35±21.01 | 28.55±18.90 | **<0.001** |
| AST(mmol/L) | 26.32±43.65 | 25.17±12.17 | 25.99±17.78 | 26.07±16.61 | 27.05±15.99 | **0.001** |
| Cr(umol/L) | 84.73±59.63 | 80.47±32.87 | 79.86±32.25 | 79.18±38.95 | 84.21±45.65 | **0.002** |
| BUN(mmol/L) | 4.92±2.61 | 4.82±2.14 | 4.77±2.26 | 4.70±2.14 | 5.09±2.66 | **<0.001** |
| FPG(mmol/L) | 5.39±0.75 | 5.97±0.68 | 6.16±0.79 | 6.56±1.17 | 9.15±3.62 | **<0.001** |
| HbA1c(%) | 6.18±0.96 | 5.95±0.69 | 5.76±0.73 | 5.77±1.02 | 6.70±2.01 | **<0.001** |

Data were presented as mean ± standard deviation or n (%). FPG/HbA1c ratio: fasting plasma glucose to glycated hemoglobin ratio; BMI: Body Mass Index; PIR: Poverty Impact Ratio; SBP: Systolic blood pressure; DBP: Diastolic blood pressure; LDL: Low-density lipoprotein; HDL: High-density lipoprotein; TC: Total Cholesterol; TG: Triacylglycerol; AST: Aspartate transaminase; ALT: Alanine aminotransferase; BUN: Blood urea nitrogen; Scr: Serum creatinine; FPG: Fasting Plasma Glucose; HbA1c: Glycosylated hemoglobin.

Supplementary Table 2. Sensitivity Analysis of the Effect of FPG/HbA1c ratio on CVD and All-Cause Mortality at Established Thresholds

|  | Sensitivity-1  (HR, 95%CI, P-value) | Sensitivity-2  (HR, 95%CI, P-value) | Sensitivity-3  (HR, 95%CI, P-value) | Sensitivity-4  (HR, 95%CI, P-value) |
| --- | --- | --- | --- | --- |
| CVD mortality |  |  |  |  |
| FPG/HbA1c ratio  < 1.080 | 0.455 (0.147, 0.854) 0.003 | 0.172 (0.056, 0.533)  0.002 | 0.343 (0.105, 0.962)  0.024 | 0.321 (0.109, 0.952)  0.043 |
| FPG/HbA1c ratio  ≥ 1.080 | 2.692 (1.303, 5.560)  0.007 | 2.562 (1.284,5.775)  0.003 | 2.155 (0.877, 5.369)  0.061 | 2.956 (1.643, 5.361)  <0.001 |
| P for Log-likelihood ratio | 0.002 | 0.001 | 0.031 | 0.003 |
| All-cause mortality |  |  |  |  |
| FPG/HbA1c ratio < 1.013 | 0.244 (0.061, 0.972) 0.035 | 0.193 (0.077, 0.414) <0.001 | 0.129 (0.055, 0.336) <0.001 | 0.264 (0.131, 0.532)  <0.001 |
| FPG/HbA1c ratio  ≥1.013 | 1.880 (1.113, 3.921) 0.012 | 2.423 (1.767, 3.565) <0.001 | 2.811 (1.842, 4.268)<0.001 | 3.132 (2.412, 4.203)  <0.001 |
| P for Log-likelihood ratio | 0.010 | <0.001 | <0.001 | <0.001 |

Sensitivity-1: including 7068 participants with prediabetes. Adjusted for age, gender, race, education, poverty impact ratio, drinking, smoking, systolic blood pressure, diastolic blood pressure, alanine aminotransferase, aspartate transaminase, serum creatinine, blood urea nitrogen, total cholesterol, triglyceride, high density lipoprotein, low density lipoprotein.

Sensitivity-2: including 1883 participants with hypoglycaemic drugs or insulin. Adjusted for age, gender, race, education, poverty impact ratio, drinking, smoking, systolic blood pressure, diastolic blood pressure, alanine aminotransferase, aspartate transaminase, serum creatinine, blood urea nitrogen, total cholesterol, triglyceride, high density lipoprotein, low density lipoprotein.

Sensitivity-3: including 1556 participants with cardiovascular disease. Adjusted for age, gender, race, education, poverty impact ratio, drinking, smoking, systolic blood pressure, diastolic blood pressure, alanine aminotransferase, aspartate transaminase, serum creatinine, blood urea nitrogen, total cholesterol, triglyceride, high density lipoprotein, low density lipoprotein.

Sensitivity-4: After excluding participants who died within the first two years of follow-up, 9996 participants were included. Adjusted for age, gender, race, education, poverty impact ratio, drinking, smoking, hypertension, alanine aminotransferase, aspartate transaminase, serum creatinine, blood urea nitrogen, total cholesterol, triglyceride, high density lipoprotein, low density lipoprotein.
